# Make It Less Complex: Autoencoder for Speckle Noise Removal—Application to Breast and Lung Ultrasound

**DOI:** 10.3390/jimaging9100217

**Published:** 2023-10-10

**Authors:** Duarte Oliveira-Saraiva, João Mendes, João Leote, Filipe André Gonzalez, Nuno Garcia, Hugo Alexandre Ferreira, Nuno Matela

**Affiliations:** 1 Instituto de Biofísica e Engenharia Biomédica, Faculdade de Ciências, Universidade de Lisboa, 1749-016 Lisbon, Portugalnmatela@fc.ul.pt (N.M.); 2 LASIGE, Faculdade de Ciências, Universidade de Lisboa, 1749-016 Lisbon, Portugal; nrgarcia@ciencias.ulisboa.pt; 3 Critical Care Department, Hospital Garcia de Orta E.P.E, 2805-267 Almada, Portugal

**Keywords:** Speckle noise, ultrasound, deep learning

## Abstract

Ultrasound (US) imaging is used in the diagnosis and monitoring of COVID-19 and breast cancer. The presence of Speckle Noise (SN) is a downside to its usage since it decreases lesion conspicuity. Filters can be used to remove SN, but they involve time-consuming computation and parameter tuning. Several researchers have been developing complex Deep Learning (DL) models (150,000–500,000 parameters) for the removal of simulated added SN, without focusing on the real-world application of removing naturally occurring SN from original US images. Here, a simpler (<30,000 parameters) Convolutional Neural Network Autoencoder (CNN-AE) to remove SN from US images of the breast and lung is proposed. In order to do so, simulated SN was added to such US images, considering four different noise levels (σ = 0.05, 0.1, 0.2, 0.5). The original US images (N = 1227, breast + lung) were given as targets, while the noised US images served as the input. The Structural Similarity Index Measure (SSIM) and Peak Signal-to-Noise Ratio (PSNR) were used to compare the output of the CNN-AE and of the Median and Lee filters with the original US images. The CNN-AE outperformed the use of these classic filters for every noise level. To see how well the model removed naturally occurring SN from the original US images and to test its real-world applicability, a CNN model that differentiates malignant from benign breast lesions was developed. Several inputs were used to train the model (original, CNN-AE denoised, filter denoised, and noised US images). The use of the original US images resulted in the highest Matthews Correlation Coefficient (MCC) and accuracy values, while for sensitivity and negative predicted values, the CNN-AE-denoised US images (for higher σ values) achieved the best results. Our results demonstrate that the application of a simpler DL model for SN removal results in fewer misclassifications of malignant breast lesions in comparison to the use of original US images and the application of the Median filter. This shows that the use of a less-complex model and the focus on clinical practice applicability are relevant and should be considered in future studies.

## 1. Introduction

Ultrasound (US) imaging is considered the most-affordable clinical imaging technique [1]. In addition, it is a safe and effective method for diagnosing a number of medical conditions [2,3].

US imaging has been used, for example, in the diagnosis of Breast Cancer (BC). BC is the most-commonly diagnosed type of cancer worldwide [4]. BC screening programs, where diagnosis usually occurs before symptoms appear, are based on mammography [5]. Despite its positive impact on mortality rates, a study found a reduction of almost 30% [6], and there are some downsides to it such as, for example, the existence of false positives and false negatives [7]. Moreover, it is also known that mammography sensitivity decreases for women having denser breasts [8]. US imaging plays a role in overcoming this specific disadvantage [9,10].

COVID-19 has, as of March 2023, infected over 759-million people worldwide, causing almost 7-million deaths [11]. The portability of Point-Of-Care US (POCUS) emerges as a safe and viable alternative for evaluating COVID-19 since it does not require patient transportation to clinical facilities [12]. Besides, it was found that higher lung US scores are related to severe stages of the disease, meaning that POCUS may play an important role in patient risk stratification [13]. Moreover, the use of POCUS in respiratory failure has shown excellent performances in terms of the diagnosis’s speed and accuracy [14].

Despite all the benefits, US images typically show Speckle Noise (SN), which limits contrast resolution and decreases lesion conspicuity. As a consequence, diagnostic interpretation is impaired [15,16]. SN usually presents itself as granularity in otherwise clean US images, but its aspect is dependent on the characteristics of both the machinery used and the analyzed tissues. This Speckle appearance occurs when US waves emitted by the source are reflected on a surface or tissue that is irregular [17]. What happens is that, after the waves are emitted, they interact with different tissues and organs (that may be irregular) and are reflected into the sensor with different phases. This results in random constructive and destructive synergies between these waves, which creates a random granular pattern called Speckle [16].

Mathematically, SN appears multiplied by the US signal resulting from wave reflection on the tissue. Therefore, for that reason, the SN level is directly dependent on the local pixel intensity of the area where it occurs. Consequently, SN not only increases image blurriness, but also decreases its contrast [18].

Therefore, the signal received by the US machinery is composed of a useful signal and SN. In fact, Speckle is a multiplicative type of noise [15], but it is composed of a multiplicative and additive fraction [18]. For that reason, it can be mathematically modeled through Equation (1).

(1) O(x,y)=S(x,y)M(x,y)+A(x,y)

where *(x,y)* are the 2D coordinates of the US image, *O* is the observed signal, *S* is the original signal, *M* is the multiplicative component of the noise, and *A* is the additive component of the noise. Since the multiplicative component is usually much more significant than the additive component, the latter is usually disregarded [18].

Multiplicative SN can be shown as a Rayleigh distribution, following the probability density function found in Equation (2):

(2) $$p\left(x|\sigma \right)=\frac{x}{\sigma^{2}}exp\left(\frac{-x^{2}}{2*\sigma^{2}}\right)$$

where *x* represents a random variable with a Rayleigh distribution, and the σ parameter represents the noise level, as it decreases lesion conspicuity [19].

The presence of SN represents a problem when assessing medical US images. For that reason, different efforts have been made to remove this type of noise while preserving image characteristics.

Given what was discussed above, filtering is an essential step for image analysis. The main challenge is to remove SN without changing important features of the image [15].

There are several classical approaches to remove SN, based on filtering techniques. The Mean and Median filters are two commonly used approaches. While the first one replaces the central pixel of the neighborhood being analyzed by the average value in that neighborhood, the latter replaces it with the Median. Since the Mean filter considers all pixel values at the analyzed location, it results in detailed blurring, which does not occur with the Median filter. This difference occurs because the Median filter only considers the intermediate pixel value, which also helps to preserve edges [20]. The Wiener filter, which operates based on local variance values, applies more smoothing where the variance is lower and has also been successfully used for removing SN [20,21]. Adaptive-Mean filters are also widely used for SN removal [22]. The Lee filter is one such example. When applying it, smoothing is specifically performed in regions with low variance, rather than high variance. This approach preserves the edges present in the image, as edges are typically characterized by higher variance regions [15]. This selective smoothing can present itself as a problem since it disregards the presence of SN near the edges. The Kuan filter, also used for SN removal, converts the multiplicative noise into additive noise. It is very similar to the Lee filter, but it uses a different weighting function [23].

A study from Khan et al. [22] compared the capability of several filters in the task of SN removal. This comparison used the Root-Mean-Squared Error, Peak Signal-to-Noise Ratio (PSNR), Speckle Suppression Index, Standard Deviation-to-Mean Ratio, and Structural Similarity Index Measure (SSIM) as performance metrics. Their analysis showed that, for variance values of pixel intensities below one, the filters that were generally more robust in removing SN from the US images were the Median, Lee, and Kuan filters.

Moving forward to novel approaches, Artificial Intelligence (AI) has made its way into the medical field, and noise removal is no exception. Recent research has been aiming to reduce the effect of SN in medical US images using Deep Learning (DL).

The work of Mishra et al. [24] consisted of using AI for SN removal of liver US images. Their model was a Convolutional Neural Network (CNN) made up of a series of three ResNet [25] blocks (with skip connections), besides having a convolutional block at the beginning and at the end of the network. The authors found that their DL model outperformed all the classical techniques for despeckling US [26,27,28,29], in terms of the PSNR and SSIM. Despite the robust results that this approach achieved, the ground-truth Despeckled data were obtained through state-of-the-art approaches, which means that, instead of learning how to obtain clean data from noisy US images, the model was learning how to mimic those approaches or an ensemble of them.

Another study [30] aimed to remove SN from US images. To do that, a pre-trained Residual Network was used and tested on US images with simulated added SN and on natural images. The variance of the simulated noise assumed values of: $\sigma^{2}=$ 0.02; 0.04; 0.06; 0.08; and 0.1. Model performance on natural images was assessed with the PSNR and SSIM, while for US images, a non-reference quality metric was used—Naturalness Image Quality Evaluator (NIQE) [31]. The authors compared their performance with that of classical filters (for example, Mean, Median, Lee, Kuan) and found that the model outperformed all filters in terms of both the PSNR and SSIM for all noise levels. Similar results were found for the NIQE metric when evaluating SN suppression in US images. The authors also aimed to evaluate model performance in removing naturally occurring SN that occurs in clinical US images. They found, once again, that their model achieved a higher NIQE value than that of classic filters, showing that the DL approach might be better at removing naturally occurring SN in US images. One of the downsides of their evaluation is that NIQE is primarily designed for evaluating the quality of natural images. While it can provide a measure of perceived quality for general images, it may not be the most-appropriate metric to assess the quality of US images.

A group of researchers aimed to test five different networks in the task of SN removal: a dilated convolution autoencoder, two U-shaped networks (one with batch normalization and another with batch re-normalization), a generative adversarial network, and a CNN-Residual Network [32]. In order to do that, they first added simulated SN to their medical US images with four different noise levels (σ = 0.1, 0.25, 0.5, 0.75). Then, they trained several classic DL architectures and tested them on the task of removing the added SN with two different test sets. Besides comparing the performance of the networks, in terms of the PSNR and SSIM, the authors also compared them to classical filtering techniques (including the Median, Mean, Lee, and Kuan filters). It was found, considering the first dataset, that the autoencoder was the architecture with the best PSNR for the three highest noise levels. Besides, in terms of the SSIM, it was a U-net that outperformed all the others. Moreover, the authors found that, in general, the proposed DL techniques outperformed classical methods in terms of removing simulated added SN.

Although the studies described here demonstrated that DL models outperform several classical filters, they did not focus on the clinical relevance of their models. Additionally, it is worth noting that most of the proposed architectures had more than 500,000 parameters, with all of them surpassing 150,000 parameters [24,30,32]. This elevated parameter count directly corresponds to heightened computational resource requirements, encompassing increased processing power, memory, and time demands. Training and deploying such models can be resource-expensive and costly. Furthermore, point-of-care US is frequently used in scenarios characterized by limited memory and processing power. The real-time or online application of complex models can be compromised in such contexts. Finally, larger models require more energy consumption, which, in turn, increases carbon emissions, which have a detrimental impact on the environment [33].

The novelty of our work focused on simplifying DL approaches for SN reduction in medical US images towards a more-environmentally friendly, cost-effective, and resource-efficient model. Moreover, a classification task was also explored, considering the importance of studying the impact of naturally occurring SN removal in clinical practice.

The remainder of the paper is organized as follows. Section 2 starts by exploring the data used in this study and their division into different sets. It continues by deeply describing the constructed models, their variations, and the parameter configuration for training. Section 3 presents the results. Section 4 discusses the obtained findings while comparing our work to the ones presented in Section 1. Finally, Section 5 addresses the general findings of the work, draws attention to the limitations associated with the followed methodology, and points out possible directions of future work.

## 2. Materials and Methods

### 2.1. Data

The data used in this study consisted of breast and lung US images.

For noise removal, the breast dataset [34] is composed of US images of three different categories: benign lesions; malignant lesions; and normal. In total, there are 780 US images. However, some of them present annotations over the lesions and, for that reason, could not be used for algorithm development. All the US images with annotations near the edges were manually cropped so that each image used to develop the algorithm was clean. Given that, 340 benign, 180 malignant, and 131 normal US images were used. The data division of the breast US images was performed randomly, with 70% of the US images being used for training and 30% for validation.

The lung US dataset used in this study, acquired at Hospital Garcia de Orta, Almada, Portugal, is composed of US images of 31 COVID-19 patients. Each patient was screened in two anterior and two lateral regions of each hemithorax. Some patients were also screened for three posterior regions of the hemithorax. For each of these regions, a 5 s video was recorded. With the rationale of increasing the amount of data without compromising data variability, Frames 1 and 100 were extracted from each video. As performed for the breast US images, all the lung US images containing annotations were cropped. The final sample consisted of 574 lung US images. The data division was performed like for the breast US images, dividing the original sample into 70% for training and 30% for validation while guaranteeing that the frames of the same video were kept within the same set.

Given the limitation of the nonexistence of US images without naturally occurring SN, the initial approach used in this work was focused on removing simulated SN added to the original US images. The noise was added to every image in both datasets through Equation (1), using the distribution presented in Equation (2). Four different noised copies of the original dataset were made, each with different σ values (0.05, 0.1, 0.2, 0.5). Besides that, the Median and Lee filters were applied to both the noised and original US images. The employment of the Lee filter was done through the following Equation (3):

(3) $$P_{ij}=\bar{K}+W*\left(C-\bar{K}\right)$$

where *P* is the new intensity of the smoothed pixel. $\bar{K}$ is the mean intensity of the kernel *K* where the filter is being applied, *C* is the center pixel, and *W* is the weighting function defined in Equation (4):

(4) $$W=\frac{{\sigma_{k}}^{2}}{{\sigma_{k}}^{2}+\sigma_{i}^{2}}$$

where ${\sigma_{k}}^{2}$ is the variance inside the kernel window and ${\sigma_{i}}^{2}$ is the variance of the image. In regions where smoothing does not occur, the output of the filter is $\bar{K}$ [15].

Finally, to allow for fair comparisons, original, noised, and filtered US images were normalized with the rationale of their pixel intensity falling within the range [0, 255].

### 2.2. Model

#### 2.2.1. Architecture

The developed model was created with the rationale of removing the SN presented in the US image that was given to it as the input while using a clean reconstructed image as a target. Considering that, a Convolutional Neural Network Autoencoder (CNN-AE) presented itself as a promising line of work to pursue. In order to learn, the model asks for a pair of input–target output. Considering the aim of this work, the pair input–target output was composed of the original US images with simulated added SN noise and the original US images, respectively. An autoencoder [35] is composed of two main blocks: an encoder and a decoder. The encoder tries to learn meaningful representations of the input data while trying to diminish the dimensions by which this representation is described [36]. The decoder, on the other hand, attempts to, with the information of the encoded representation, reconstruct the input with the characteristics presented in the target output. Considering that, in this work scenario, what separates the input from the target output is the presence of SN. Therefore, SN is the meaningless information that the model learns to disregard when reconstructing the encoded information into the target output.

**Figure 1:**
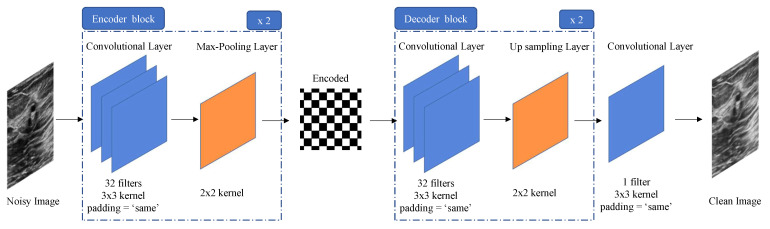
Architecture of the model, consisting of two sequential encoder and decoder blocks. This architecture results in a number of parameters that varies from 320 to 9248 inside the blocks and ends with 289 parameters in the last convolutional layer.

The model CNN-AE (Figure 1) starts with an input layer of 536 × 232, following the common lung US image’s shape. The encoder, which is the first block of the developed model, is composed of two alternating convolutional and max-pooling layers. The convolutional layers have 32 filters, a kernel size of 3 × 3, a ReLU activation function, and the option “same” for padding. This means that the output of the layer will have precisely the same size as the input. The max-pooling layers have a kernel size of 2 × 2, with no padding being made. This means that, after the first “convolution—max-pooling” block, the input representation has a size of (268,116,32). Therefore, the encoded representation that appears after the second “convolution—max-pooling” block has a size of (134,58,32).

The decoder part of the model is also composed of alternating layers. While the convolutional layers remain equal to what was seen for the encoder, the max-pooling layers are substituted by upsampling layers, also with a 2 × 2 kernel size, without padding. The rationale behind this architecture is to mirror the operations performed in the encoding portion of the model. The upsampling layers do precisely that by recovering, step-by-step, the original size of the input. This means that, after the first upsampling layer, the data representation has a size of (268,116,32), and the ultimate output has, as expected, dimensions of (536,232,32). Finally, the last layer of the model, which results in the decoded output, is a convolutional layer with one filter, a kernel size of 3 × 3, and a linear activation function. This last layer allows the data representation to match the exact dimensions of the input in all dimensions—(536,232,1).

The weights were initialized using Xavier initialization, and the model, developed in Python 3.8, was trained for 1000 epochs using the Adam Optimizer (β1 = 0.9, β2 = 0.999, ϵ = $10^{-7}$, and a learning rate of 0.001)—running in an NVIDIA GeForce RTX 3060 Graphics Processing Unit board (GPU). Figure 1 outlines the developed model, and Table 1 describes each layer.

#### 2.2.2. Training

After defining the architecture, different models were created with distinct training data, and their performance was assessed with the validation set. Results were obtained for each of the following models:

1. Full model: Four models were trained, each with a different noise level of the Rayleigh distribution (σ = 0.05, 0.1, 0.2, 0.5). The noised US images from the breast and lung datasets were used as inputs, while the corresponding original US images were defined as the target output. Each model had a validation set with the respective noise level.
2. Breast model: Four models were trained, each with a different noise level of the Rayleigh distribution (σ = 0.05, 0.1, 0.2, 0.5). The noised US images from the breast dataset were used as inputs, while the corresponding original breast US images were defined as the target output. Each model had a validation set with the respective noise level.
3. Lung model: Four models were trained, each with a different noise level of the Rayleigh distribution (σ = 0.05, 0.1, 0.2, 0.5). The noised US images from the lung dataset were used as inputs, while the corresponding original lung US images were defined as the target output. Each model had a validation set with the respective noise level.
4. Full model with original US images: The input of the model consisted of original breast and lung US images of the training sets. The original US images filtered using the Lee filter were used as the targets.

The first three models aimed to verify, through different combinations of training sets, if the CNN-AE was capable of satisfactorily removing simulated added SN.

For each of these three models, their performance was compared with the performance of the Median and Lee filters. Finally, the full model with original US images was developed to verify if the CNN-AE could learn filtering characteristics and reproduce them faster.

To evaluate models’ performance, the SSIM [37], which measures the similarity between two US images, and the PSNR, which approximates what humans perceive as reconstruction qualities [38], were used. Each of these metrics were computed using Equations (5) and (6), respectively:

(5) $$SSIM\left(x,y\right)=\frac{\left(2\mu_{x}\mu_{y}+c1\right)\left(2\sigma_{xy}+c2\right)}{\left(\mu_{x}^{2}+\mu_{y}^{2}+c1\right)\left(\sigma_{x}^{2}+\sigma_{y}^{2}+c2\right)}$$

where $\mu_{x}$ and $\mu_{y}$ are the mean pixel intensity value in US images *x* and *y*, respectively; $\sigma_{x}^{2}$ and $\sigma_{y}^{2}$ are the variance of US images *x* and *y*; $\sigma_{xy}$ is the cross-correlation between *x* and *y*; and c1,c2 are constants to avoid problems with the division by a very small denominator.

(6) $$PSNR=\frac{10log_{10}{\left(peak_{val}\right)}^{2}}{MSE}$$

where $peak_{val}$ is the maximum pixel intensity present on the image and MSE is the Mean-Squared Error between the analyzed US images.

Each model configuration performance was assessed with these two metrics by comparing the output of the respective noised validation set with the original US images (higher values correspond to better performance in removing SN). Besides that, the same metrics were also computed for the output of the filters applied to the noised validation sets.

### 2.3. Real-World Applicability

While these noise-removal techniques tend to show good results in improving image quality, it is important to understand if they have a practical result in real-world applications. For that reason, we proposed the development of a CNN to differentiate between benign and malignant lesions in breast US images. Then, we aimed to compare its performance when using US images before and after naturally occurring SN removal.

In order to do that, the US images from the breast dataset were divided into two classes of lesions: benign and malignant. Each image pixel intensity histogram was computed, and the top and bottom 5% of intensity values were clipped. After that process, the US images’ pixel intensities were normalized to the range [0, 255]. Finally, all US images were resized to dimensions 256 × 256, considering the usual shape in this type of research. After that, the model was trained using 5-fold cross-validation, while keeping the class proportion among the different folds.

The CNN architecture was based on a work by Muduli et al. [39] and had already been used by our team in the task of benign/malignant lesion differentiation [40]. The network architecture is shown in Table 2.

**Figure 2:**
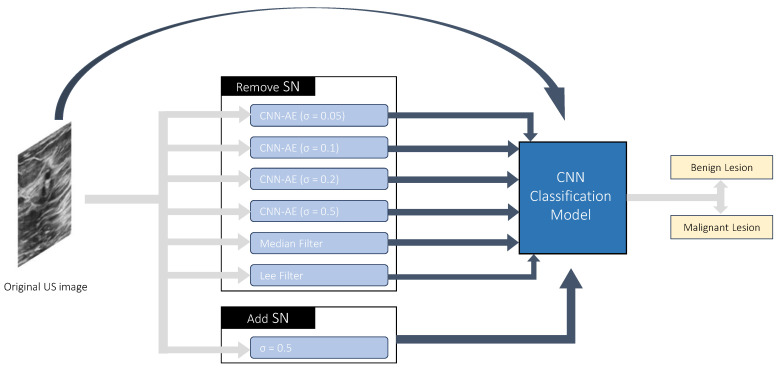
Diagram showing the eight different inputs that fed the developed Convolutional Neural Network (CNN) models for breast cancer classification: the original Ultrasound (US) images; the output of the developed full CNN-AE models (σ=0.05,0.1,0.2,0.5) when the original US images were used as the input; the output of the Median filter applied to the original US images; the output of the Lee filter applied to the original US images; the original US images with a simulated added Speckle Noise (SN) level of 0.5.

Eight different models were trained using different inputs (Figure 2). The original US images were used for one configuration. The output of the developed full CNN-AE models (σ=0.05,0.1,0.2,0.5) when the original US images were used as input derived four more model configurations. Besides that, the Median and Lee filters were applied to the original US images, and the resulting data were used to train two more model configurations, one for each filter used. Finally, to check if this model was robust to the presence of noise, US images with the simulated noise level of 0.5 were used to train the model. The models’ performance was evaluated through the following metrics: accuracy (Equation (7)); sensitivity (Equation (8)); specificity (Equation (9)); Positive Predictive Value (PPV) (Equation (10)); Negative Predictive Value (NPV) (Equation (11)); F1-score (Equation (12)); and Matthews Correlation Coefficient (MCC) (Equation (13)).

(7) $$Accuracy=\frac{TP+TN}{TP+TN+FP+FN}$$

where *TP* is “True Positive”, *FN* is “False Negative”, *TN* is “True Negative”, and *FP* is “False Positive”.

(8) $$Sensitivity=\frac{TP}{TP+FN}$$

(9) $$Specificity=\frac{TN}{TN+FP}$$

(10) $$PPV=\frac{TP}{TP+FP}$$

(11) $$NPV=\frac{TN}{TN+FN}$$

(12) $$F1-score=\frac{2*PPV*Sensitivity}{PPV+Sensitivity}$$

(13) $$MCC=\frac{TP*TN-FP*FN}{\sqrt{\left(TP+FP)(TP+FN)(TN+FP)(TN+FN\right)}}$$

## 3. Results

### 3.1. Speckle Noise Removal

Table 3, Table 4 and Table 5 present the SSIM metric for three different model configurations: full, lung, and breast models, at the four different noise levels. For each noise level, three SSIM values are presented: the comparison between filter outputs (Median and Lee) with the original US images and the comparison between CNN-AE outputs and the original US images.

Similar to what was seen for the SSIM, Table 6, Table 7 and Table 8 present the PSNR results for the full, lung, and breast models, respectively. This value was computed, as for the SSIM, between the output of the respective validation sets for each model and for each noise level. In order to compare the performance of the developed models with classical approaches, this metric was also computed for the output of each validation set when the Median and Lee filters were applied to it.

The SSIM results of the full model presented in Table 3 show that, as the noise level increased, there was a decrease in the similarity between the US images that the CNN-AE output and the original US images. For noise levels of σ=0.05,0.1,0.2,0.5, the SSIM values were 0.97, 0.95, 0.93, and 0.86, respectively. Across noise levels of σ=0.05,0.1,0.2,0.5, the CNN-AE consistently outperformed the Median filter (0.92, 0.88, 0.80, 0.62) and the Lee filter (0.92, 0.89, 0.83, 0.68). Even though the tendency was the same, it seems that the CNN-AE was more robust to noise variations than the Median and Lee filters. From σ = 0.05 to σ = 0.1, the SSIM of the CNN-AEs decreased by 0.02, with values of 0.04 and 0.03 being observed for the Median and Lee filter, respectively. When transitioning from σ = 0.1 to σ = 0.2, the decrease seen in the SSIM of the CNN-AE was 0.02 once again, while for the Median and Lee filters, it was 0.08 and 0.06, respectively. Finally, when moving to the highest noise level, the SSIM of the CNN-AEs decreased by 0.07 and the SSIM of the Median and the Lee filters decreased by 0.18 and 0.15, respectively.

For the lung model and for the breast models, in Table 4 and Table 5, respectively, the results were similar. As the noise level increased from σ = 0.05 to σ = 0.5, there was a general decrease in the SSIM. For the lung model, the SSIM values were 0.95, 0.94, 0.91, and 0.84; for the breast model, the values were 0.98, 0.97, 0.94, and 0.90. Additionally, the CNN-AE outperformed the Lee filter for every noise level. It was also possible to observe that the absolute decrease was larger for the Lee filter than for the lung and breast models for σ=0.05,0.1,0.2,0.5, respectively. Similar conclusions can be drawn when comparing the absolute SSIM decrease for the CNN-AE vs. the Median filter.

Finally, looking at the PSNR results in Table 6, Table 7 and Table 8 for every noise level and for every model configuration, the CNN-AE outperformed the Median and Lee filters. As expected as well, the PSNR values decreased with increasing noise levels.

To determine if the proposed CNN-AE model could replicate the Lee filter’s method of removing noise, the full model with the original US images was created. The results showed that the output of the CNN-AE had a SSIM of 0.98 when compared to the output of the Lee filter, showing that the developed model could replicate standard filtering techniques. Considering the data used for validation input, nearly 370 US images, the noise removal from the entire dataset was approximately 1008 s for the filter and 3 s for the CNN-AE. Therefore, not only was the developed model capable of reproducing the results of standard filtering techniques, but it could also do so in a fraction of the time.

### 3.2. Real-World Applicability

The evaluation of the clinical importance of SN removal was performed by creating a CNN that uses breast US images to differentiate between benign and malignant breast lesions. Following that, eight configurations of that model were tested under the exact same conditions (model architecture, folds’ configuration). The only difference was the noise in each image: (1) original US images; (2) denoised original US images (CNN-AE σ = 0.05); (3) denoised original US images (CNN-AE σ = 0.1); (4) denoised original US images (CNN-AE σ = 0.2); (5) denoised original US images (CNN-AE σ = 0.5); (6) denoised original US images (Median filter); (7) denoised original US images (Lee filter); (8) original US images with simulated added SN (σ = 0.5).

Table 9 shows that the same US images processed in different ways resulted in different performances of the CNN model. For the sensitivity, the use of the denoised US images (CNN-AE—σ = 0.2; CNN-AE—σ = 0.5 and the Lee filter) achieved the best results, around 0.760, whereas the noised US images resulted in the lowest value of sensitivity, approximately 0.712. Similar results were obtained for the NPV, with the denoised CNN-AE—σ = 0.5 US images obtaining the highest value, approximately 0.876, immediately followed by the CNN-AE—σ = 0.2 and the Lee filter (0.875). On the contrary, the noised US images led to the lowest value, around 0.855. Regarding specificity and the PPV, the original US images yielded the highest results, approximately 0.959 and 0.903, respectively, while the lowest results were obtained using the US images denoised by the Lee filter, around 0.906 for specificity and 0.830 for the PPV. The highest F1-score was obtained using the denoised US images (CNN-AE—σ = 0.1), approximately 0.802. The lowest F1-score was obtained using the noised US images, approximately 0.767. Considering the MCC and accuracy, the original US images registered the highest results, approximately 0.718 and 0.873, respectively. The lowest results were obtained using the noised US images, around 0.666 for the MCC and 0.849 for the accuracy.

When considering the various noise levels employed in the CNN-AE models, it is worth noting that σ = 0.1 consistently yielded the best results in terms of the F1-score, MCC, and accuracy. For sensitivity and the NPV, σ = 0.2 and σ = 0.5 achieved the highest results, while σ = 0.05 led to the lowest value among the CNN-AE models. The opposite trend was observed for specificity and the PPV. Overall, the noise levels influenced the different metrics in distinctive ways. Similar observations can be made for the Median and Lee filters, with the first only outperforming the second for specificity and the PPV.

## 4. Discussion

### 4.1. Speckle Noise Removal

From the results presented in Section 3.1, it was shown that, as the noise level of the developed CNN-AE models decreased, both the SSIM and PSNR increased their values, indicating a better performance of the model in removing simulated added SN. Additionally, all CNN-AE models outperformed the Median and Lee filters for both metrics at the same noise level. It was also shown that one of the developed CNN-AE models was approximately 300-times faster than the Lee filter. This clearly demonstrates the significant computational advantage of employing this DL architecture, which holds substantial importance when considering the creation of algorithms to aid in clinical practice.

Given the studies introduced and reviewed in Section 1, it is possible to make a fair comparison between the work developed here and the one presented in [32]. Just like in the work presented here, the authors tested the capability of removing simulated added SN from medical US images using DL approaches and compared it to the performance of classical filters, using the SSIM and PSNR in two different test sets. The main difference with the work of these authors is that we propose a DL approach that is less complex when compared with the architectures introduced by them. Most of the proposed architectures have more than 500,000 parameters, while our network has below 30,000 parameters. Considering that it is more computationally efficient, the proposed solution translates into a more-environmentally friendly, cost-effective, and resource-efficient model.

In the study [32], five different network architectures were proposed: a dilated convolution autoencoder; two U-shaped networks (one with batch normalization and another with batch re-normalization); a generative adversarial network; and a CNN-residual network. In contrast, our approach focused on simplicity, employing an autoencoder with a lower number of parameters with only two convolution layers in both the encoder and decoder stages. Regarding the incorporation of noise, Karaoğlu et al. [32] added simulated SN with values of σ=0.1,0.25,0.5,0.75. However, we recognized that a level of 0.75 would lead to an image with so much noise that it would not be realistic in a clinical environment. For that reason, we adjusted the noise levels to σ=0.05,0.1,0.2,0.5. While the authors of [32] developed, for each architecture, one model that encapsulated US images of all noise levels, here, more-specialized models were developed, with one model per noise level.

Comparing the performance across the same noise levels, it becomes evident that the CNN-AE models approached the best PSNR results achieved by Karaoğlu et al. [32]. Specifically, at σ=0.1, their best PSNR was 34.32, while our full, lung, and breast models achieved 33.19, 33.33, and 34.96, respectively. For σ=0.5, their best PSNR was 31.45, while our full, lung, and breast models achieved 29.23, 28.42, and 29.11. When looking not only for their best results, but for all the PSNR values obtained by their five models when σ=0.1, it is possible to verify that the CNN-AE full and lung models outperformed three and four of their five proposed networks, respectively. Moreover, the breast model outperformed their five networks. In their second test set, for σ=0.1, the full and lung models outperformed three of the five proposed networks, while the breast model performed better than four of them. When analyzing the same values for σ=0.5, the full, lung, and breast models outperformed three models in both the first and second test sets. Regarding the SSIM, in the context of the first test set, the five proposed networks generally outperformed the CNN-AE models at both σ=0.1 and σ=0.5. In their second test set, for σ=0.1, the full model outperformed two of the five proposed networks, while the breast model outperformed four of them. For σ=0.5, all five networks outperformed the full and lung models, whereas the breast model outperformed three of the five networks.

Overall, we showed that a DL network with a fraction of the number of parameters can perform better than more-complex architectures. Although some of the proposed networks by [32] achieved better results in terms of the PSNR and SSIM, we must note that they are less-environmentally friendly, cost-effective, and resource-efficient than the network proposed here. Moreover, care should be taken when considering that higher PSNR and SSIM values directly translate into clinical value, as discussed in the following section.

### 4.2. Real-World Applicability

In the previous section, it was demonstrated that the developed model is accurate at removing the simulated added SN from the US images. Although scientifically interesting, it does not assess the removal of the naturally occurring SN of original US images. To do so, the trained models were tested with original US images with the aim of removing the naturally occurring SN of the US images. However, the evaluation of the quality of those output US images is complex. Considering the lack of non-reference quality metrics, which are necessary for such an evaluation, the authors of [30] proposed the use of NIQE as a quality metric for evaluating the removal of the naturally occurring SN of US images. However, NIQE was designed to evaluate the quality of natural images, and its reliability on medical US images is still unknown.

Nonetheless, it should be remembered that the goal of improving image quality, and in particular the removal of SN, is to improve diagnostic decisions. Accordingly, the previously mentioned CNN that differentiates malign from benign lesions in breast US images was created.

For the particular task described here, the MCC is a relevant metric, since it is useful for assessing binary classification performance, especially in imbalanced datasets. The best MCC was obtained when using the original US images as the input. The same was observed for accuracy, while for the F1-score, only the denoised (CNN-AE with σ=0.1) US images outperformed the original US images. The CNN-AE (σ=0.1) was also the best model for the MCC, accuracy, and F1-score compared to the other CNN-AE models. Moreover, for these metrics, all the CNN-AE models outperformed the filters. Besides, using noised data as the input to the classifier resulted in the worst results for these three metrics. For specificity and PPV, a similar pattern was found, with the use of original US images resulting in the best performances. On the contrary, for sensitivity and the NPV, almost all denoised US images outperformed the original ones. Furthermore, as the noise level decreased, the CNN-AE exhibited improved results for specificity and the PPV. Conversely, it showed lower results for sensitivity and the NPV. These are interesting results since sensitivity and specificity evaluate the ability of the model to correctly detect malignant and benign lesions among those who actually are malignant and benign lesions, respectively. Furthermore, the PPV and NPV are the proportions of malignant and benign results in diagnostic tests that are true malignant and true benign results, respectively. In clinical practice, the non-detection of a malignant lesion (false negative) is more concerning than its incorrect detection (false positive), suggesting that sensitivity and the NPV are the two most-relevant metrics for clinical practice. This considered, the application of denoised methods led to better results, demonstrating that the removal of SN may be important for the correct detection of malignant breast lesions. The CNN-AE (σ=0.05) and the Median filter were consistently less effective. Although the CNN-AE (σ=0.1) outperformed the CNN-AE (σ=0.2), the CNN-AE (σ=0.5), and the Lee filter in terms of the F1-score, MCC, and accuracy, its sensitivity and NPV were lower. The CNN-AEs (σ=0.2 and σ=0.5) attained the most-favorable outcomes, surpassing the Lee filter in terms of the NPV and exhibiting comparable sensitivity. As a result, these models emerged as the optimal choices for clinical application.

It is worth noting that the highest PSNR and SSIM values do not translate into better performance of the CNN classification model. In fact, the highest-performing CNN-AE model in regard to the PSNR and SSIM aligns with the lowest-performing CNN-AE model in terms of added clinical value (lowest sensitivity and NPV).

The findings may suggest that, although the task of removing SN from the original US images led to the worst MCC, F1-score, and accuracy results, in comparison to the original US images, its application is still relevant for clinical practice as it improves sensitivity and the NPV, which are the most-relevant metrics for reducing the misdiagnosis of malignant breast lesions.

## 5. Conclusions

Motivated by the goal of improving diagnostic capability using US images and considering the lack of practical AI-based solutions for SN removal, we proposed a DL model to remove SN from US images with a 5× to 20× lower number of parameters compared with other proposed DL approaches [24,30,32]. All CNN-AE models achieved better results, in terms of the SSIM and PSNR, when compared with the Median and Lee filters. We also showed that our model was less affected by the increment in the noise level than the filters. The results gave us a hint that simplicity may be the solution.

The novelty of this study was the investigation of the removal of naturally occurring SN from original US images for a real-world application. Based on a CNN classification model that differentiates malignant from benign breast lesions, we tested the impact of SN removal in such a clinical task. Our results showed that removing SN decreased the MCC, F1-score, and accuracy of the CNN classification model. However, considering that the correct detection of a malignant lesion is the most-essential task in clinical practice, sensitivity and the NPV should be considered as targets for evaluating the developed model. Considering this, the CNN-AEs (σ=0.2 and σ=0.5), which were not the best models in terms of the PSNR and SSIM, may be considered the most-appropriate models for the clinical task considered in this study.

Overall, our exploratory study stands as a way to argue that every AI application for solving medical problems must focus on its application to the real world. In the case of SN removal, please note that:

1. CNN-AE’s performance compares to the Lee filter in terms of sensitivity and the NPV, but without compromising diagnostic accuracy, needing parameter tuning, or being time-consuming.
2. A low-parameter CNN-AE is able to achieve high performance and is more computationally efficient and cost-effective than more-complex models.
3. For models trained with US images with simulated added SN as the input, it is essential to test them with the original US images to evaluate their applicability in the real world.

When considering image quality improvements, researchers should focus on its benefits to clinical practice. Here, we demonstrated that, although the CNN-AE (σ=0.05) achieved the best results in terms of the SSIM and PSNR, it did not display a clear benefit in real-world applicability. On the other hand, the CNN-AE trained with higher noise levels showed an increased capability to correctly identify malignant breast lesions, which is extremely important in clinical practice. As a matter of fact, the CNN-AE generally outperformed the classification model trained with the original US images in terms of both sensitivity and the NPV.

As a final note, the effort that is being made in the task of SN noise removal might not necessarily contribute to a distinct added value to clinical practice. In this medical domain, the current studies have focused on removing simulated added SN, often overlooking the challenge of removing naturally occurring SN. Moreover, there is no focus on the relevance of removing such naturally occurring SN in terms of medical diagnosis. Our study showed that the SSIM and PSNR metrics do not directly translate into added clinical value. For that reason, future steps in this area of research should study the clinical impact of removing naturally occurring SN present in US images. Only by doing that will the DL models actually add value to real-world clinical practice.

## Figures and Tables

**Table 1:** Architecture summary of the Convolutional Neural Network Autoencoder (CNN-AE).

Layer (Activation Function)	Kernel Size	Channels	Output Shape	# of Param
Input Layer	-	-	(536, 232, 1)	0
Convolutional Layer (ReLU)	3 × 3	32	(536, 232, 32)	320
Max-Pooling Layer	2 × 2	-	(268, 116, 32)	0
Convolutional Layer (ReLU)	3 × 3	32	(268, 116, 32)	9248
Max-Pooling Layer	2 × 2	-	(134, 58, 32)	0
Convolutional Layer (ReLU)	3 × 3	32	(134, 58, 32)	9248
Upsampling Layer	2 × 2	-	(268, 116, 32)	0
Convolutional Layer (ReLU)	3 × 3	32	(268, 116, 32)	9248
Upsampling Layer	2 × 2	-	(536, 232, 32)	0
Convolutional Layer (Linear)	3 × 3	1	(536, 232, 1)	289

**Table 2:** Architecture summary of the Convolutional Neural Network (CNN) for cancer classification.

Layer	Kernel Size	Channels	Output Shape	# of Param
Input Layer	-	-	(256, 256, 1)	0
Convolutional Layer	9 × 9	16	(256, 256, 16)	1312
Batch Normalization Layer	-	-	(256, 256, 16)	64
ReLU	-	-	(256, 256, 16)	0
Max-Pooling Layer	2 × 2	-	(128, 128, 16)	0
Convolutional Layer	7 × 7	32	(128, 128, 32)	25,120
Batch Normalization Layer	-	-	(128, 128, 32)	128
ReLU	-	-	(128, 128, 32)	0
Max-Pooling Layer	2 × 2	-	(64,64,32)	0
Convolutional Layer	5 × 5	64	(64, 64, 64)	51,264
Batch Normalization Layer	-	-	(64, 64, 64)	256
ReLU	-	-	(64, 64, 64)	0
Max-Pooling Layer	2 × 2	-	(32, 32, 64)	0
Convolutional Layer	3 × 3	128	(32, 32, 128)	78,356
Batch Normalization Layer	-	-	(32, 32, 128)	512
ReLU	-	-	(32, 32, 128)	0
Max-Pooling Layer	2 × 2	-	(16, 16, 128)	0
Flatten Layer	-	-	32,768	0
Fully Connected Layer	-	128	128	4,194,432
Dropout Value (0.5)	-	-	-	0
Fully Connected Layer	-	1	1	129

**Table 3:** SSIM results for the full model.

Full Model—SSIM			
**Noise Level (σ)**	**Median Filter**	**Lee Filter**	**CNN-AE**
0.05	0.92 ± 0.05	0.92 ± 0.05	**0.97 ± 0.01**
0.1	0.88 ± 0.05	0.89 ± 0.05	**0.95 ± 0.02**
0.2	0.80 ± 0.06	0.83 ± 0.06	**0.93 ± 0.02**
0.5	0.62 ± 0.08	0.68 ± 0.06	**0.86 ± 0.04**

**Table 4:** SSIM results for the the lung model.

Lung Model—SSIM			
**Noise Level (σ)**	**Median filter**	**Lee filter**	**CNN-AE**
0.05	0.87 ± 0.02	0.87 ± 0.01	0.95 ± 0.01
0.1	0.83 ± 0.02	0.84 ± 0.02	0.94± 0.01
0.2	0.75 ± 0.04	0.78 ± 0.03	0.91 ± 0.01
0.5	0.57 ± 0.06	0.65 ± 0.05	0.84 ± 0.03

**Table 5:** SSIM results for the breast model.

Breast Model—SSIM			
**Noise Level (σ)**	**Median Filter**	**Lee Filter**	**CNN-AE**
0.05	0.96 ± 0.01	0.97 ± 0.01	0.98 ± 0.00
0.1	0.93 ± 0.02	0.94 ± 0.02	0.97 ± 0.01
0.2	0.85 ± 0.04	0.88 ± 0.03	0.94 ± 0.01
0.5	0.66 ± 0.06	0.71 ± 0.06	0.90 ± 0.02

**Table 6:** PSNR results for the full model.

Full Model—PSNR (dB)			
**Noise Level (σ)**	**Median Filter**	**Lee Filter**	**CNN-AE**
0.05	31.05 ± 4.04	31.24 ± 4.16	36.60 ± 2.62
0.1	26.11 ± 3.65	26.45 ± 3.83	33.19 ± 3.49
0.2	21.27 ± 2.86	21.62 ± 2.96	31.62 ± 2.83
0.5	16.83 ± 2.36	17.15 ± 2.44	29.23 ± 2.79

**Table 7:** PSNR results for the lung model.

Lung Model—PSNR (dB)			
**Noise Level (σ)**	**Median Filter**	**Lee Filter**	**CNN-AE**
0.05	29.14 ± 2.28	29.19 ± 2.30	33.48 ± 1.74
0.1	25.28 ± 2.61	25.50 ± 2.66	33.33 ± 1.82
0.2	21.31 ± 2.41	21.63 ± 2.49	31.03 ± 2.28
0.5	17.23 ± 2.09	17.56 ± 2.17	28.42 ± 2.50

**Table 8:** PSNR results for the breast model.

Breast Model—PSNR (dB)			
**Noise Level (σ)**	**Median Filter**	**Lee Filter**	**CNN-AE**
0.05	32.75 ± 4.48	33.07 ± 4.58	36.42 ± 3.25
0.1	26.55 ± 3.97	26.96 ± 4.16	34.96 ± 3.30
0.2	21.41 ± 3.25	21.80 ± 3.38	31.02 ± 3.38
0.5	16.45 ± 2.39	16.77 ± 2.45	29.11 ± 3.07

**Table 9:** Evaluation metrics of the classification of benign and malignant breast lesions, for each of the different model inputs (original, denoised (CNN-AE—σ = 0.05), denoised (CNN-AE—σ = 0.1), denoised (CNN-AE—σ = 0.2), denoised (CNN-AE—σ = 0.5), denoised (Median filter), denoised (Lee filter), noised σ = 0.5)). Sensitivity, specificity, Positive Predictive Value (PPV), Negative Predictive Value (NPV), F1-score, Matthews Correlation Coefficient (MCC), accuracy.

Evaluation Metrics							
**Model Input**	**Accuracy**	**Sensitivity**	**Specificity**	**PPV**	**NPV**	**F1-Score**	**MCC**
Original	0.873 ± 0.020	0.716 ± 0.035	0.959 ± 0.012	0.903 ± 0.028	0.861 ± 0.016	0.799 ± 0.033	0.718 ± 0.045
Denoised CNN-AE 0.05	0.866 ± 0.025	0.716 ± 0.065	0.947 ± 0.020	0.888 ± 0.039	0.863 ± 0.026	0.784 ± 0.048	0.704 ± 0.056
Denoised CNN-AE 0.1	0.871 ± 0.021	0.749 ± 0.044	0.939 ± 0.010	0.867 ± 0.023	0.873 ± 0.021	0.802 ± 0.034	0.713 ± 0.047
Denoised CNN-AE 0.2	0.864 ± 0.025	0.759 ± 0.037	0.921 ± 0.027	0.845 ± 0.044	0.875 ± 0.018	0.798 ± 0.036	0.700 ± 0.054
Denoised CNN-AE 0.5	0.864 ± 0.023	0.759 ± 0.045	0.921 ± 0.017	0.840 ± 0.034	0.876 ± 0.022	0.796 ± 0.035	0.698 ± 0.052
Denoised Median Filter	0.853 ± 0.018	0.744 ± 0.046	0.912 ± 0.036	0.841 ± 0.051	0.870 ± 0.019	0.781 ± 0.025	0.682 ± 0.038
Denoised Lee Filter	0.854 ± 0.019	0.760 ± 0.033	0.906 ± 0.033	0.830 ± 0.050	0.875 ± 0.013	0.788 ± 0.025	0.684 ± 0.040
Noised 0.5	0.849 ± 0.016	0.712 ± 0.042	0.924 ± 0.021	0.843 ± 0.037	0.856 ± 0.017	0.767 ± 0.027	0.666 ± 0.037

## Data Availability

The lung ultrasound data were provided by Hospital Garcia de Orta (Lisboa, Portugal) and are not publicly available. The breast ultrasound data, Accessed on 18 January 2023 can be found here: https://www.kaggle.com/datasets/aryashah2k/breast-ultrasound-images-dataset.
